# Supplementary material for: Histological Patterns and Mammographic Presentation of Invasive Lobular Carcinoma Show No Obvious Associations
Source: Cancers (Basel). 2024 Apr 24;16(9):1640. doi: 10.3390/cancers16091640 (PMC11083920; doi:10.3390/cancers16091640)
Supplement: Supplementary file 1 [file cancers-16-01640-s001.zip › cancers-2968004-supplementary.pdf]

Table S1: Clinical and pathological features of the tumors assessed

	All	Radiology review	Pathology review	Radio-Pathology correlation
Number	146	141	136	132
Age: mean (range) years	63.8 (40-91)	64.1 (40-91)	64.1 (40-91)	64.3 (40-91)
Laterality L/R	74/72	71/70	68/68	66/66
Histological grade 1/2/3	3/133/10	3/129/9	3/123/8	3/122/7
(y)pT categories				
pT1mi - pT1a	4	3	2	1
pT1b	14	14	13	13
pT1c	59	57	57	55
pT2	49	48	47	46
pT3	19	18	17	17
pT4b	1	1	0	0
pN categories				
pN0	87	84	82	79
pN1	35	34	34	33
pN2	11	11	10	10
pN3	11	10	8	8
pNx	2	2	2	2
